# Effect of High Glucose Concentration on Collagen Synthesis and Cholesterol Level in the Phenotypic Modulation of Aortic Cultured Smooth Muscle Cells of Sand Rat (*Psammomys obesus*)

**DOI:** 10.1080/15438600490489793

**Published:** 2004

**Authors:** S. Aouichat Bouguerra, Y. Benazzoug, F. Bekkhoucha, M. C. Bourdillon

**Affiliations:** 1 Nutrition and Metabolism Physiology (LBPO) P.O. Box 32 USTHB Algiers Algeria; 2 Extracellular Matrix (LBCM) FSB USTHB Algiers Algeria; 3 EA 3740 Université Claude Bernard Lyon-1 Faculté de Médecine RTH Laënnec Lyon Cedex France; 4 Central Hospital of the Army Ain Naâdja Algiers Algeria

## Abstract

To simulate diabetic conditions, the effects of high glucose concentration on collagen synthesis and cholesterol level in cultured aortic smooth muscle cells of *Psammomys* were investigated. For collagen biosynthesis, smooth muscle cells (SMCs) were incubated in synthetic proliferative phase and in postconfluent phase with ^3^H-proline. Cellular cholesterol was determined by enzymatic method. Under high glucose concentration, the results showed morphological modifications characterized by morphometric cellular, nuclear, and nucleolar changes. In biochemical studies, the authors observed an increase of free and esterified cellular cholesterol as well as of total proteins, collagen biosynthesis, and *α*1 (I+III) and *α*2 (I) chains of collagen contained in the SMCs and in the extracellular matrix. These results showed the sensitivity of *Psammomys* aortic SMCs to high glucose concentration and would constitute an interesting cellular model to study atherosclerosis pathogeny in experimental diabetes.


*Experimental Diab. Res.*, 5:227–235, 2004
Copyright © Taylor and Francis Inc.
ISSN: 1543-8600 print / 1543-8619 online
DOI: 10.1080/15438600490489793

Taylor & Francis
Taylor & Francis Group

### Effect of High Glucose Concentration on Collagen Synthesis and Cholesterol Level in the Phenotypic Modulation of Aortic Cultured Smooth Muscle Cells of Sand Rat (*Psammomys obesus*)

**S. Aouichat Bouguerra,[1] Y. Benazzoug,[2] F. Bekkhoucha,[4] and M. C. Bourdillon[3]**

[1]*Nutrition and Metabolism Physiology (LBPO) and* [2]*Extracellular Matrix (LBCM),FSB, USTHB, Algiers Algeria*
[3]*EA 3740 Université Claude Bernard Lyon-1, Faculté de Médecine RTH Laënnec, Lyon Cedex, France*
[4]*Central Hospital of the Army, Ain Naâdja, Algiers, Algeria*

**To simulate diabetic conditions, the effects of high glucose concentration on collagen synthesis and cholesterol level in cultured aortic smooth muscle cells of *Psammomys* were investigated. For collagen biosynthesis, smooth muscle cells (SMCs) were incubated in synthetic proliferative phase and in postconfluent phase with $^3$H-proline. Cellular cholesterol was determined by enzymatic method. Under high glucose concentration, the results showed morphological modifications characterized by morphometric cellular, nuclear, and nucleolar changes. In biochemical studies, the authors observed an increase of free and esterified cellular cholesterol as well as of total proteins, collagen biosynthesis, and $\alpha 1$ (I + III) and $\alpha 2$ (I) chains of collagen contained in the SMCs and in the extracellular matrix. These results showed the sensitivity of *Psammomys* aortic SMCs to high glucose concentration and would constitute an interesting cellular model to study atherosclerosis pathogeny in experimental diabetes.**

**Keywords** Cholesterol; Collagen, $\alpha 1$ (I + III) and $\alpha 2$ (I) Chains; Glucose; *Psammomys*; Smooth Muscle Cells

Received 26 May 2003; accepted 30 May 2004.
The authors express their thanks to Mr. Y. Bouguerra, C.E.O., and Mr. S. A. Gharsa, Operation Director, of Cimet for their help in the styling of the manuscript and Mr. K. Gernigon for all his services.
Address correspondence to S. Aouichat Bouguerra, Nutrition and Metabolism Physiology, P.O. Box 32, FSB, USTHB, Algiers, Algeria.

The smooth muscle cells (SMCs) of the media are directly involved in atherosclerosis pathogeny [1–3]. Biochemically, the major and initial event of the atherosclerosis process is related to oxidized low-density lipoprotein (LDL) [4], to SMCs migration from media to intima [5, 6], and to lipid deposit in SMCs and macrophages, which turn into foam cells [4, 7].

In the case of diabetics, atherosclerosis development occurs earlier and in more severe form [8, 9]. It is established that diabetes induced various defects in extracellular matrix [10]. Robert and Robert [11] have described quantitative and qualitative alterations of the level of total collagen. These variations would imply a decrease of collagen solubility [12] and the premature ageing of diabetic connective tissue [13].

Originally from mesenchyma, the arterial SMC is highly involved in atherosclerosis pathogeny [5, 14]. Spontaneously, or under the effect of various stimuli, it activates and changes its phenotype from a contractile sessile state to synthetic proliferative state [3, 15]. Then it enters into repeated divisions and synthesizes the extracellular matrix in an anarchic manner [16].

The object of this study is the cultured aortic SMCs of *Psammomys*, which develops a non–insulin-dependent diabetes similar to human diabetes when under hypercaloric diet [17]. *Psammomys* appears to be an interesting model because during diabetes, it develops degenerative complications of small and big vessels [18]. Thus, the study of cultured aortic SMCs of *Psammomys* has motivated our choice.

First, we explored the morphometry of great cellular and nuclear axes and nucleolus numeration, then the biosynthesis of total proteins and collagen as well as the free and the esterified fractions of cholesterol in cultured aortic SMCs of in contractile and synthetic states. We have also analyzed, by vertical electrophoresis and densitometry, the collagen $\alpha 1$ (I + III) and $\alpha 2$ (I) chains. Secondly, we investigated the effects of high glucose concentration during 4 passages for the 2 states of cultured SMCs, to simulate the hyperglycemia that characterizes the diabetic state.

### MATERIALS AND METHODS

### Animals

This study was carried out on 7 *Psammomys* aged between 3 and 6 months and weighing 80 to 100 g. When trapped, animals were maintained in conditions previously described by Marquié and colleagues [19] and Aouichat Bouguerra and colleagues [20]. *Psammomys* is native of Beni Abbes region in southwest of Algeria (wilaya of Béchar, 30°7 north latitude and 2°10 west longitude). This diurnal species belongs to Muridae family and Gerbillidae subfamilly; its life expectancy is 3 years and eats exclusively halophil plants (rich in water and minerals) from Chenapodiaceae family, especially *Traganum nudatum, Suaeda mollis*, and *Salsola foetida* [21]. Animals were fed a natural plant diet (50 g/day) during 6 months and the daily caloric intake was 20 kcal. After the 6th month of experiments, they were killed.

### Analytical Techniques

Animals were bled from the retro-orbital venous plexus as previously described by Aouichat Bouguerra and colleagues [20]. Blood glucose and cholesterol were measured by the enzymatic method using a test kit of Boehringer. Blood insulin was determined by radioimmunoassay using CIS test kit (Oris Indus).

### Aortic Smooth Muscle Cell Culture

Cell culture technique was used according to Ross [1] and modified by Bourdillon and colleagues [2]. Explants were obtained from thoracic aorta; they were prepared after removing adventitia by collagenase action at 0.1% (type IA; Sigma, USA) and incubated in Dulbecco's modified Eagle's medium (DMEM) (Gibco, USA), supplemented with 10% fetal calf serum (Sigma, USA), penicillin (50 IU/mL), streptomycin (50 $\mu$g/mL) and L-glutamine at 200 mM (Gibco, USA). The explants were maintained at 37°C under air-$CO_2$ (95%–5%) atmosphere until they reached confluence. Then, SMCs were trypsinized (0.08% of trypsin; Gibco, USA) and subcultured. In this experiment, the SMCs were used in the 4th passage in the synthetic state ($10^6$ cells/mL) and contractile state ($2.5 \times 10^6$ cells/mL) in view of compared morphometric and biochemical study (cholesterol assay, total proteins, collagen biosynthesis, densitometric analysis of $\alpha 1$ (I + III) and $\alpha 2$ (I) collagen chains and level of type I and type III collagen).

### Morphometric Measurements

In proliferative state for the synthetic phenotype and at confluency for the contractile phenotype, the medium was eliminated from the patches and the SMCs were washed with phosphate-buffered saline (PBS) at 10% then fixed in aqueous bath and colored with May-Grunwald-Giemsa. Morphometric analysis was carried out on 100 measurements of cellular and nuclear axes as well as nucleolus numeration.

### Cellular Cholesterol

Free and esterified cholesterol were assessed according to the method of Salé and colleagues [22]. The enzymes used (Boehringer Mainheïm) were Nocardia cholesterol oxidase (45 IU/mg), horseradish peroxidase (250 IU/mg), and ester hydrolase cholesterol (55 IU/mg). After trypsin action, SMCs of each flask were picked up by 1.1 mL of 10% Hank's saline solution. Lipids were extracted according to the method of Folch and colleagues [23] by chloroform-methanol mixture (2:1; *v*/*v*). The assay of cholesterol fraction was carried out, after evaporation, on dry extracts taken up in ethanol.

### Total Proteins and Collagen Biosynthesis

Radiolabeled proline incorporation (L-5-$^3$H-proline, specific activity 20 Ci/mmol [Isotopchim, France] at 10 $\mu$Ci/mL, for 24 hours in medium culture containing 10 $\mu$g/mL of ascorbic acid without fetal calf serum) defined biosynthesis of total proteins and collagen. Extracellular and intracellular fractions were submitted to 2 successive dialyses, at 4°C for 24 hours, respectively against running water and 0.5 M of acetic acid. A first aliquot was taken to assess the rate of total protein biosynthesis.

To determine the biosynthesis of total collagen, samples (medium and cells) were treated at the end of the incubation by a dialysis pepsination (200 $\mu$g/mL of pepsin; Biochemical) against 0.5 mM acetic acid at 4°C for 24 hours [24]. The radioactivity was measured in liquid scintillation counter and results were expressed in cpm/$10^6$ cells.

### Glucose Effects on Smooth Muscle Cells in Culture

For our experiments on the influence of high glucose concentration, SMCs were incubated in proliferative and confluent phases in a culture medium containing 5% fetal calf serum and glucose at extraphysiological concentrations (15 mM

equivalent to 3 g/L) during 4 passages compared to normal conditions with 5 mM glucose equivalent to 1 g/L, to observe the effects of glucose on cells size, cholesterol concentration, collagen biosynthesis and $\alpha 1$ (I + III) and $\alpha 2$ (I) collagen chains and ratio of type III collagen/type I collagen.

### Densitometric Analysis of $\alpha 1$ (I + III) and $\alpha 2$ (I) Collagen Chains and Collagen Type

After pepsination, the medium or extracellular compartment was lyophilized and resuspended in buffer solution (Tris-HCl 0.05 M, sodium dodecyl sulfate (SDS) 14%, bromophenol blue 0.05%, and EDTA 2 mM). The collagen samples were separated by vertical electrophoresis on 10% polyacrylamide gel (SDS-PAGE) in reducing conditions with 0.25% $\beta$-mercaptoethanol according to the procedure of Laemmli [25]. Gels were stained in colored solution (Coomassie blue 0.025%, propanol 25%, acetic acid 10%) for 24 hours and discolored in 10% acetic acid; $\alpha 1$ and $\alpha 2$ collagen chain levels were evaluated by densitometry. At the end of this step, the gel was dried for 3 hours at 80°C and the $\alpha$ chains of collagen were quantified by excision of each $\alpha$ band. The radioactivity was eluted in hydrogen peroxide at 12% for 24 hours measured by liquid scintillation and expressed in cpm/$10^6$ cells.

The radioactivity recovered in type I and type III collagens was quantified by numeric integration and the level was evaluated by the ratio radioactivity in the medium/total radioactivity in the medium and cells.

### Statistical Analysis

Data in Tables 1 to 3 were expressed as the mean (± SD) for all parameters. Glycemia, lipidemia, morphometric study, collagen chains, type I and III collagens and collagen III/collagen I ratio were analyzed statistically with Student's $t$ test. Insulin, cellular free and esterified cholesterol, cellular and extracellular total protein, and total collagen were analyzed with distribution-free Mann and Whitney $U$ test.

**TABLE 1**
Evolution of body weight and plasma biochemical parameters in *Psammomys* under hypocaloric diet (halophilus plants)

| | Natural diet | | |
|---|---|---|---|
| | 0 Month | 6 Months | |
| Body weight (g) | 77.7 ± 4.4 | 92.2 ± 3.9 | $P < .02$ |
| Glucose (mg/100 mL) | 60.2 ± 5.1 | 69.0 ± 7.0 | $P < .02$ |
| Insulin ($\mu$U/mL) | 25.2 ± 5.9 | 37.3 ± 7.1 | $P < .02$ |
| Esterified cholesterol (mg/100 mL) | 34.3 ± 4.7 | 45.2 ± 5.5 | $P < .04$ |

*Note*. Animals were killed at 9 months old. Values are means ± SD for 7 animals. Significance of differences refers to 0 month. Body weight and plasma biochemical parameters in *Psammomys* under hypocaloric diet (halophilus plants). Glucose and esterified cholesterol were measured by the enzymatic colorimetric method. Blood insulin was determined by radioimmunoessay. At 6 months, the statistical analysis was slightly significant compared to the control group.

**TABLE 2**
Effects of high glucose concentration on synthesis of total proteins by contractile and synthetic cultured aortic SMCs of *Psammomys obesus*e

| | cSMCs | sSMCs | |
|---|---|---|---|
| Total protein synthesis (cpm/$10^6$ cells) | | | |
| + G 5 mM | 504 ± 24 | 1748 ± 239 | $P < .004$ |
| + G 5 mM 4 P | 2197 ± 140 $P < .004$ | 4110 ± 268 | $P < .004$ |

*Note*. Values are means ± SD of 6 determinations. Significance tests refered to control group and evaluated between the extracellular compartment (medium) and the intracellular compartment (cells). Exposure of aortic SMCs to high glucose during 4 passages indicated an increased production of the total proteins (*** $P < 0.004$).

### RESULTS

### Biochemical Study

The biochemical parameters are shown in Table 1. These parameters indicated that glucose, insulin, and free and esterified cholesterol were modified under hypocaloric diet after the 6th month ($P < .04$). Also, the body weight was significantly increased ($P < .02$).

### Morphometric Study

In the proliferative phase, aortic SMCs of *Psammomys* were in the synthetic state and presented a polygonal aspect. At confluency and postconfluency, they became contractile and were spindle-shaped (Figure 1).

Morphometric results are indicated in Figure 2. They showed that great cellular axes were more important in cSMCs than in sSMCs: 50.6 ± 6.5 $\mu$m against 47.2 ± 5.2 $\mu$m ($P < .01$). The great axes of nucleus were increased in sSMCs with 21.6 ± 1.9 $\mu$m against 16.5 ± 2.2 $\mu$m ($P < .01$). Nucleolus number was also statistically higher in sSMCs (3.2 ± 0.9) than in cSMCs (1.9 ± 0.7) ($P < .005$).

Important modifications were observed when SMCs were incubated in culture medium containing high glucose concentration (15 mM) during 4 passages. Thus, in contractile and synthetic cells the respective values of maximal length were reached 70.8 ± 5.2 $\mu$m and 64.7 ± 6.2 $\mu$m ($P < .001$). The nuclear length was also higher compared to the control group and this increase was observed essentially in sSMCs as compared

**TABLE 3**

Effect of high glucose concentration (15 mM) on the level of type I and type III collagens contained in the medium of cultured aortic SMCs of *Psammomys* and on the ratio of type III/type I collagens

| | Coll III (%) | | Coll I (%) | | Coll III/Coll I | |
|---|---|---|---|---|---|---|
| cSMCs | $7.3 \pm 0.5$ | | $11.0 \pm 0.8$ | | $0.67 \pm 0.06$ | $P < .01$ |
| sSMCs | $26.6 \pm 1.9$ | $P < .001$ | $43.2 \pm 2.0$ | $P < .0001$ | $0.62 \pm 0.04$ | |
| cSMCs + G 4P | $45.0 \pm 3.1$ | $P < 10^{-5}$ | $53.6 \pm 3.2$ | $P < 2 \times 10^{-5}$ | $0.84 \pm 0.01$ | $P < .01$ |
| cSMCs + G 4P | $54.5 \pm 4.4$ | $P < .0001$ | $84.2 \pm 4.6$ | $P < 10^{-6}$ | $0.64 \pm 0.04$ | $P < 3 \times 10^{-5}$ |

*Note*. Collagen types were evaluated when $\alpha 1$ (I + III) and $\alpha 2$ (I) chains were separated on SDS-PAGE electrophoresis. The incorporation of $^3$H-proline in the medium was expressed as cpm/$10^6$ cells and the levels of type III (Coll III) and type I collagen (Coll I) were determined as described in Materials and Methods. Values are means ± SD of 6 determinations. The ratio coll III/coll I of all experimental conditions are shown. This ratio was higher in contractile state than in synthetic state ($^{**}P < .01$). Under high glucose concentration, a pronounced increase of this ratio in contractile state was noted and statistical test was significant as compared to synthetic state ($^{****}P < .0001$) and control group ($^{**}P < .01$).

with cSMCs, 30.7 ± 4.6 versus 21.1 ± 1.9 $\mu$m ($P < .01$). Nucleolus number was increased in sSMCs ($P < .01$), with 3.7 ± 1.2 against 2.7 ± 0.9 in cSMCs.

### Cholesterol Level

In Figure 3, the results indicated that the free cholesterol was higher in synthetic state than in contractile state; the corresponding values were 9.9 ± 1.7 $\mu$g against 4.9 ± 0.6 $\mu$g per $10^6$ cells with statistical differences ($P < .004$). The esterified cholesterol was also more increased in favor of the synthetic state with 2.4 ± 1.1 $\mu$g/$10^6$ cells against 1.5 ± 0.4 $\mu$g/$10^6$, but the statistical difference was lower ($P < .02$).

As compared with the control groups, the incubation of SMCs in culture medium containing high glucose concentration (15 mM) during 4 passages showed an increase of free and esterified cholesterol ($P < .004$). This increase was less significant in synthetic state and respective concentrations of free and esterified cholesterol were 25.9 ± 2.8 $\mu$g/$10^6$cells ($P < .004$) and 13.0 ± 0.9 $\mu$g/$10^6$ cells ($P = .004$).

### Biosynthesis Study

The results given in Table 2 indicated that the contractile state of SMCs presented a global protein synthesis equal to 504 ± 24 cpm/$10^6$ cells. Compared to this result, global synthesis in the synthetic state was determined as 1748 ± 239 cpm/$10^6$ cells ($P < .004$). The total collagen concentration in the contractile and the synthetic state (Figure 4) was represented respectively by 12.6% ± 0.7% and 39.5% ± 3.3% ($P < .004$). In the 2 phenotypic states, the proline radiolabel in the collagen was higher in medium than in cells (Figure 5) ($P < .004$). The effect of high glucose concentration during 4 passages (Table 2) showed that the synthesis rates of total proteins and collagen increased significantly. Thus, labeling with L-5-$^3$H-proline allowed to record a global protein synthesis of

**Synthetic SMC (sSMC)** **Contractile SMC (cSMC)**

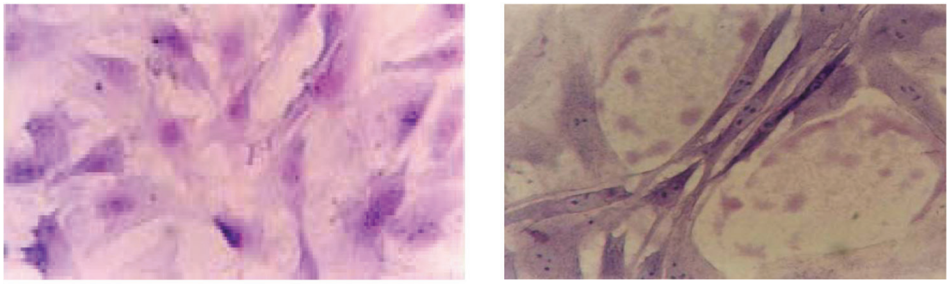

**FIGURE 1**

Microscopic appearance of cultured SMCs of *Psammomys* in the contractile and the synthetic state (G × 200). The cells were fixed and stained in May-Grunwald-Giemsa. In the proliferative phase, aortic SMCs were in the synthetic state and have polygonal form. In postconfluency phase, the cells were in the contractile state and they have a spindle-shaped aspect. The nucleolus number is higher in the synthetic state.

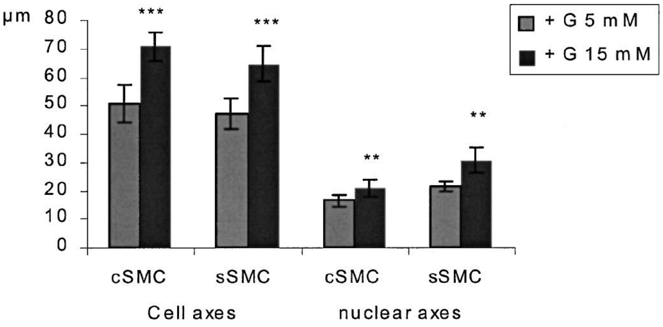

**FIGURE 2**
Morphometric study of cellular and nuclear great axes in aortic cultured cSMCs and sSMCs of *Psammomys* in 2 experimental conditions. The normal condition with glucose 5 mM (+G 5 mM) and the extraphysiological condition with glucose 15 mM (+ G 15 mM) during 4 passages. Values are means ± SD of 100 cellular, nuclear, and nucleolar measurements for each experimental group. Significance tests refer to control group and evaluated between the contractile phenotypic state (cSMCs) and the synthetic phenotypic state (sSMCs) ($^{**}P < .01$). A significant increase of all parameters with high glucose was observed ($^{**}P < .01$, $^{***}P < .001$). +G 5 mM = normal conditions with glucose added to the medium at 5 mM (equivalent to 1 g/L); +G 15 mM = extraphysiological conditions with glucose added to the medium at 15 mM (equivalent to 3 g/L) during 4 passages.

4110 ± 268 cpm/$10^6$ cells in sSMCs ($P < .004$) against 2197 ± 141 cpm/$10^6$ in cSMCs ($P < .004$). The pepsin-resistant radiolabeling corresponding to rates total collagen synthesis reached 113.0% ± 8.3% in sSMCs against 51.8% ± 2.2% in cSMCs. The statistical test was significant ($P < .004$) between the 2 states and as compared with the control group (Figure 4).

In addition, under high glucose concentration the collagen radiolabeling contained in the medium and in the sSMCs and the cSMCs indicated, respectively, 1973 ± 154 and 1027 ± 197 cpm/$10^6$ cells ($P = .004$) and 1306 ± 308 and 765 ± 108 cpm/$10^6$ cells ($P < .004$) (Figure 5). These results indicated that the medium contained more collagen than the cells for the 2 states ($P < .04$ for cSMCs; $P < .02$ for sSMCs).

The secretion rate of total collagen are shown in Figure 6. In contractile and synthetic states, the collagen secretion was equivalent respectively to 16.5% ± 0.8% and 46.6% ± 1.8%, with a significant statistical difference ($P < .004$). The total protein secretion was also higher in the synthetic state than in the contractile state (Figure 6).

The high glucose concentration during 4 passages induced a pronounced increase of the level of total collagen secretion (Figure 6). It reached 86.9% ± 5.7% in sSMCs and 34.3% ± 2.0% in cSMCs and significant as compared with the control groups ($P < .004$). When we compared the 2 phenotypic states,

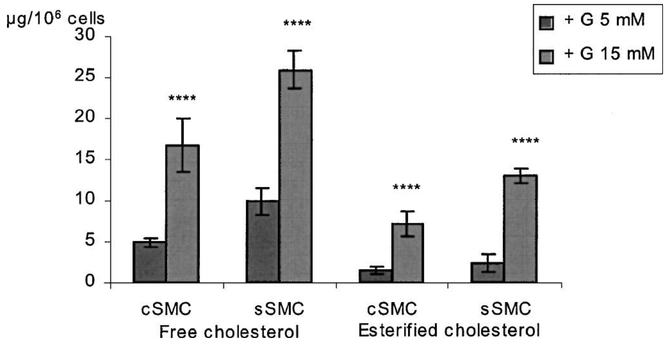

**FIGURE 3**
Determination of free and esterified cholesterol in cultured SMCs of *Psammomys* in the 2 phenotypic states studied (contractile and synthetic) and under high glucose concentration (15 mM) during 4 passages. Cell number in synthetic state: $10^6$ cells/mL and in contractile state: 2.5 × $10^6$ cells/mL. Under high glucose concentration, free and esterified cholesterol level significantly increased especially in the synthetic state. Free cholesterol concentrations were determined from standard curve of cholesterol stock solution at 1 mg/mL in ethanol. The concentration of esterified cholesterol was deduced from the difference between total cholesterol and free cholesterol concentration. Values are means ± SD of 7 determinations. Significance tests refer to control group ($^{****}P < .004$, $^{****}P < .006$). Free cholesterol concentrations were determinate from standard curve of cholesterol stock solution at 1 mg/mL in ethanol. Esterified cholesterol was deduced from between total cholesterol and free cholesterol. Significance tests refer to control group and between the 2 phenotypic states.

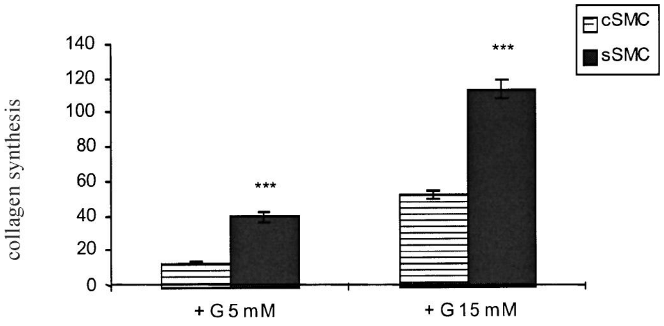

**FIGURE 4**
Effect of high glucose concentration on total collagen synthesis by aortic cultured SMCs of *Psammomys.* Values are means ± SD of 7 determinations. Significance tests refers to control group ($^{****}P < .004$). SMCs were incubated with $^3$H-proline. Radiolabeling in total collagen was determined by total pepsin resistant radiolabeling in medium and cells. Exposure of aortic SMCs to high glucose during 4 passages indicated an increased production of total collagen.

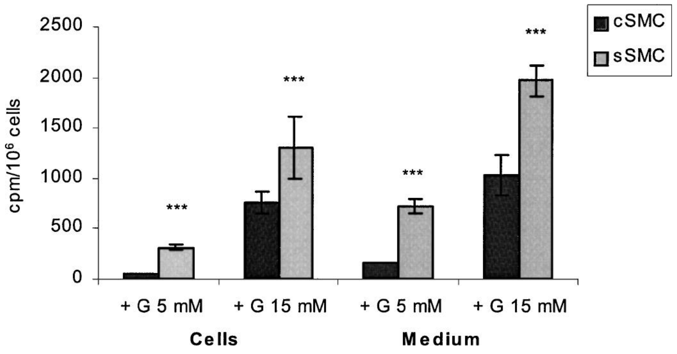

**FIGURE 5**

$^{3}$H proline radiolabeling of total collagen in medium and cells of *Psammomys* SMCs cultured in the contractile and the synthetic phenotypic states and in the 2 experimental conditions (+G 5 mM, +G 15 mM). Data are means ± SD of 6 determinations. Statistic tests between contractile and synthetic state in the 2 compartments are significant (****$P < .004$). SMCs were incubated with $^{3}$H proline. Radiolabeling in total proteins was determinated by the radiolabeling of medium and cells. Radiolabeling in total collagen was determined by total pepsin resistant radiolabeling in medium and cells. The rate of collagen corresponds to the ratio: total pepsin resistant radiolabeled/total protein synthesis. Values are means ± SD of 6 determinations. Significance differences refers to control group and evaluated between the 2 phenotypic states. The medium or the extracellular matrix was more affected by the collagen accumulation than the cells.

a significant level of secretion was observed in synthetic state ($P < .004$).

### Effect of High Glucose Concentrations on Collagen $\alpha 1$ (I + III) and $\alpha 2$ (I) Chains in Extracellular Compartment of Cultured SMCs

Collagen $\alpha 1$ (I + III) and $\alpha 2$ (I) chains contained in the medium of cSMCs and sSMCs were separated by vertical electrophoresis (Figure 7) and analysed by densitometry (Figure 8). When extraphysiological glucose concentration was added to the medium of SMCs, we observed a pronounced increase of $\alpha 1$ and $\alpha 2$ chain levels of type I and type III collagens.

The $^{3}$H-proline incorporation in the medium of cultured aortic SMCs showed that extracellular type I and type III collagens were more represented in synthetic state than in contractile state. In addition, the results revealed a substantially greater amount of type I collagen than of type III collagen contained in the medium of synthetic and contractile SMCs.

Exposure of the cells to a high glucose concentration showed a pronounced increase of type III and type I collagens in the medium as shown in Table 3. However, the evaluation of collagen III/collagen I ratio indicated significant change in the 2 phenotypic states. When the SMCs were incubated in medium containing a high normal glucose concentration (+G 5 mM) (Table 3), we noted that type III collagen/type I collagen ratio was less elevated in synthetic state (0.64 ± 0.04) than in contractile state (0.67 ± 0.06) ($P < .002$). The decrease of the ratio

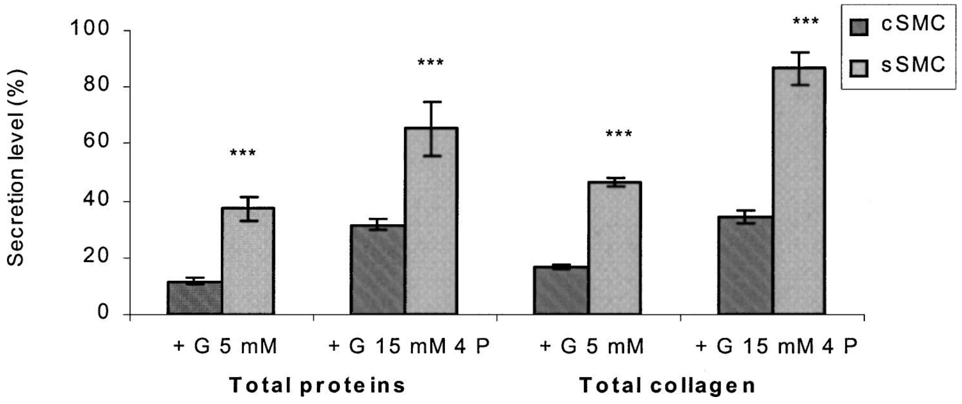

**FIGURE 6**

Total proteins and total collagen secretion by contractile and synthetic SMCs of *Psammomys* in 2 experimental conditions (+G 5 mM and +G 15 mM). Data are means of 6 determinations. Production of total protein and collagen was greater in synthetic state as compared with contractile state (***$P < .001$). Proteins (or collagen) secretion is expressed as percentage of radiolabel incorporated into extracellular compartment to total protein (or collagen) of extra- and intracellular compartments (medium + cells). Values are means ± SD of 6 determinations. Significance tests evaluated between the 2 phenotypic states and refereed to control groups indicated significant variations between contractile and synthetic states and under high glucose concentration as shown.

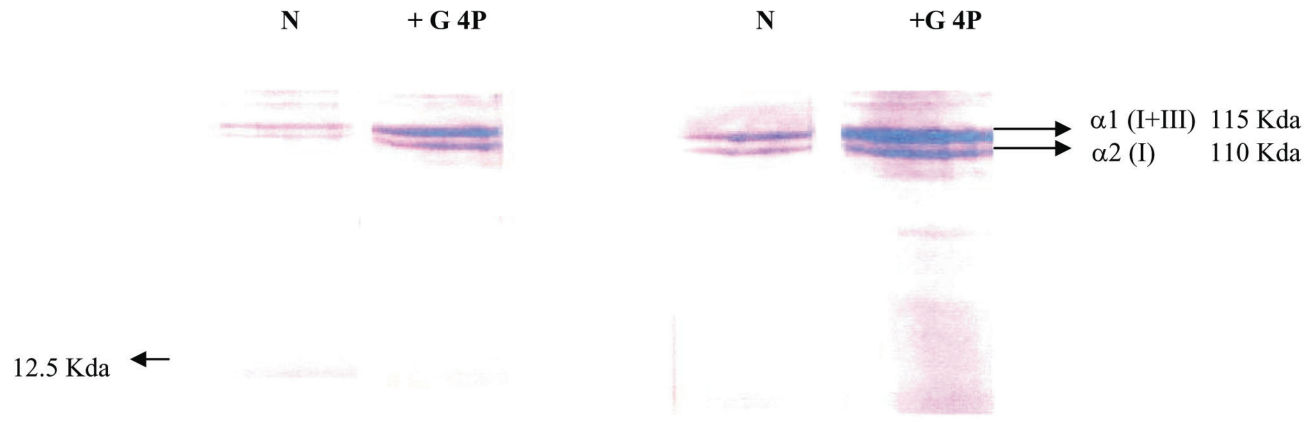

**FIGURE 7**
Separation of $\alpha 1$ (I + III) and $\alpha 2$ (I) collagen chains contained in the medium of subcultured cSMCs and sSMCs of *Psammomys* in normal experimental conditions studied (N) and in presence of 15 mM glucose in the medium during 4 passages (+G 4P). The $\alpha$ chains were analyzed after reduction with 0.25% $\beta$-mercaptoethanol by electrophoresis on 10% SDS polyacrylamide gel. Gels were stained with Coomassie blue and discolored in 10% acetic acid.

was estimated as 7.5%. Thus, these results demonstrated an increase of type I collagen production by sSMCs. The influence of high glucose concentration during 4 passages induced modifications of type III/type I collagen ratio. It showed a pronounced increase in contractile state (0.84 ± 0.01 versus 0.67 ± 0.06 in control group). In synthetic state, the ratio was slightly modified as compared to contractile state (0.64 ± 0.04 versus 0.84 ± 0.01) and decreased by 23.9%. The elevated glucose caused significant change in the collagen I/III production by the cultured sSMCs in the extracellular-matrix.

### DISCUSSION

According to works of Chamley-Campbell [7] and Campbell [26] and their colleagues, in secondary culture, aortic SMCs of *Psammomys* present 2 phenotypic forms: the synthetic form in proliferative phase and the contractile form at confluence and postconfluency. The morphometric study showed a rise of great nuclear axes and nucleolus number of sSMCs compared to cSMCs. The microscopic observation of SMCs incubated with high glucose in culture medium shows an apparent effect only on nucleolus number, significantly more increased in sSMCs. In return, morphometric approach reveals a significant raise of all parameters with higher differences for sSMCs. These modifications could be related to the functional state of the cell. Indeed, during synthetic state, according to Campbell and Chamley-Campbell [3] and Godeau [15], the SMCs present many organelles (ribosomes, endoplasmic granule dilated reticulum, mitochondria, etc.), proving increased synthesis.

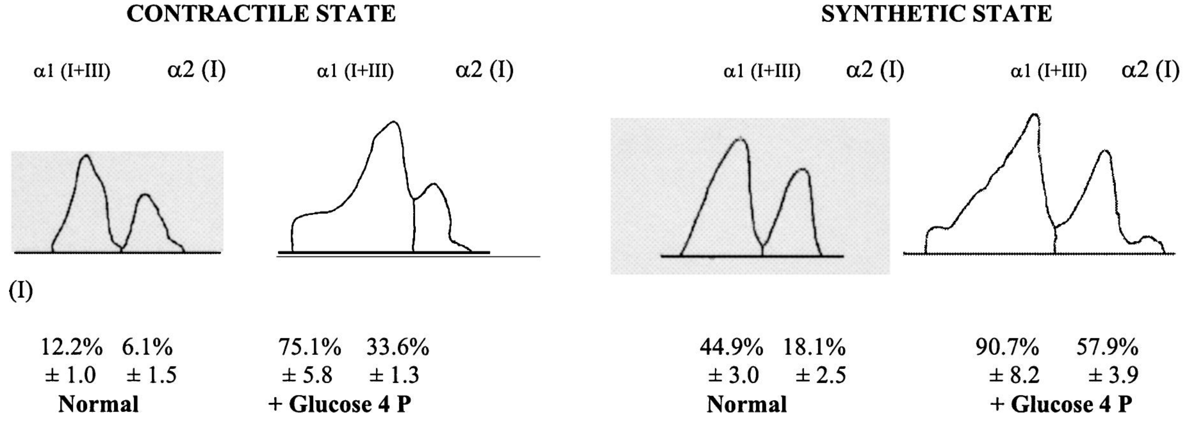

**FIGURE 8**
$\alpha 1$ (I + III) and $\alpha 2$ (I) collagen chains contained in the medium of cultured aortic SMCs of *Psammomys* were separated by vertical electrophoresis on 10% SDS-PAGE and evaluated by densitometry and was expressed in percentage of total $\alpha$ collagen chains.

In contractile state, the organelles are reduced and the SMCs are characterized by exuberance of myofilaments [5].

With regard to cholesterol metabolism, our study shows a more cholesterogenic activity in *Psammomys* synthetic sSMCs as compared to contractile state cSMCs. These results conform to those obtained by Bourdillon [27] and Dussère [28] and their colleagues in SMCs of laboratory rat. They also confirm the works of Tabacick and colleagues [29], which demonstrated in rabbit higher cholesterogenic activity of sSMCs.

Under long-term glucose effect, the level of cholesterol is more increased in cultured SMCs, which synthesized cholesterol ester probably by activation of acyl–coenzyme A (CoA) cholesterol acyl transferase and free cholesterol by activation of hydroxyl methyl glutaryl (HMG)-CoA reductase [30]. However, these observations show higher sensitivity of synthetic than contractile SMCs of sand rats *Psammomy*.

These modifications of cholesterol metabolism would result from an disturbed balance in esterified cholesterol/free cholesterol ratio, because they depend on process of uptake, biosynthesis, and cholesterol transport to the cells [31]. These variations would be a prelude to a mechanism of transformation of SMCs in foam cells rich in cholesterol ester and characteristic of atherosclerotic lesions [32, 33].

A long-term incubation of cultured aortic SMCs of *Psammomys* in high glucose concentration showed a very significant rise in biosynthesis of total proteins and collagen especially in synthetic state ($\times 2.4$ for protein, $\times 4.9$ for collagen). Thus, in the 2 states, the glucose effect in long term (4 passages) induced disturbances in biosynthesis of total proteins and collagen especially in the synthetic state. Ang and colleagues [34] observed in sSMCs of rabbit collagen and total proteins biosynthesis, respectively, 20 to 30 and 5 to 6 higher than in cSMCs. Majors and Ehrhart [35] also observed a decrease of 70% of collagen in aortic SMCs at postconfluence compared to proliferative SMCs. In the streptozotocin-diabetic rat, collagen accumulates in the medium and the fraction of insoluble collagen rises [36]. In iliac artery SMCs of rabbit, Ang and colleagues [34] observed activation of mRNA coding $\alpha 1$ (I) and $\alpha 1$ (III) procollagen chains. Equally, after experimental angioplastic surgury, Karim and colleagues [37] reported transcriptional activation of genes coding procollagen. Thus, results obtained in our study agree with those of literature and reveal, in particular, the sensitivity of *Psammomys* sSMCs. Nahman and colleagues [38] observed a slight rise of total protein biosynthesis of human mesangial cells incubated in high glucose concentration in culture medium. In human cutaneous fibroblasts incubated for 24 hours in 15 mM glucose, biosynthesis of collagen and proteins is less modified [39]. When SMCs were exposed to a high glucose concentration, they showed a significant increase of type I and type III collagen production in the 2 phenotypic states. Furthermore, we observed a pronounced increase of type I collagen production in the synthetic state. Robert and colleagues [40] explained the increase of collagen concentrations in SMCs during atherosclerotic process by type I and type III collagen neosynthesis. In another way, an increase of the production of type I collagen over type III collagen was observed in the late stages of atherosclerosis.

In diabetic macroangiopathy, the neosynthesis of collagen would cause fibrosis with consequent glycation [41, 42]. The presence of a glycated products in many human tissues in long-term contact with glucose suggests that this process increases with aging, vascular complications during diabetes, and atherosclerosis [43]. The glycation would lead, according to Robert [16], to a progressive hardening of collagen fibers and impede their renewing.

### REFERENCES

[1] Ross, R. (1971) The smooth muscle cell. II. Growth of smooth muscle in culture and formation of elastic fibers. *J. Cell Biol.,* **50,** 172–186.

[2] Bourdillon, M. C., Boisel, J. B., and Crouzet, B. (1976) Xanthomatous cell formation in vitro from rat aortic media. *Arter. Wall (Paroi artérielle).,* **3,** 101–104.

[3] Campbell, G. R., and Chamley-Campbell, J. H. (1981) Smooth muscle in atherosclerosis. *Pathology,* **13,** 424–439.

[4] Maggi, E., Perani, G., Falashi, F. Fratoni, A., Martignoni, A., Finardi, G., Stephano, P. L., Simeone, F., and Paolini, G. (1994) A autoantibodies against oxidised low density lipoproteins in patients with coronary disease. *Press. Med.,* **23,** 1158–1162.

[5] Ross, R. (1986) The pathogenesis of atherosclerosis. *N. Engl. J. Med.,* **314,** 488–500.

[6] Guyton, J. R. (1994) The arterial wall and the atherosclerosis lesion. *Curr. Opin. Lipidol.,* **5,** 376–381.

[7] Chamley-Campbell, J., Campbell, G. R., and Ross, R. (1979) The smooth muscle cell in culture. *Physiol. Rev.,* **59,** 1– 61.

[8] West, K. (1978) Epidemiology of diabetic and its vascular lesions. *Elsevier. Shanon.*

[9] Bucala, R., and Cerami, A. (1992) Advenced glycosylation: Chemistry, biology and implications for diabetes and aging. *Adv. Pharmacol.,* **23,** 1–34.

[10] Kern, P., Moczar, M., and Robert, L. (1979) Biosynthesis of skin collagens in normal and diabetic mice. *Biochem. Med.,* **30,** 189.

[11] Robert, L., and Robert, B. (1970) *Exposés. Ann. Biochimie. Med.,* **30,** 189.

[12] Kohn, R. R., and Schneider, S. L. (1989) Collagen changes in aging skin. In: *Aging and the Skin,* edited by Balin, A. K., and Kligman, A. M., pp 120–140. New York, Raven Press.

[13] Goldstein, S., and Harley, C. B. (1979) In vitro studies of age associated diseases. *Fed. Proc.,* **38,** 1862–1867.

[14] Hadjiisky, P., Bourdillon, M. C., and Grogogeat, Y. (1991) Modèles expérimentaux d'athérosclérose. Apports, limites et perspectives. *Arch. Mal. Cœur.,* **84,** 1593–1603.

[15] Godeau, G. (1990) La cellule musculaire lisse. *Revue de la veine et du lymphatique.,* **4,** 8–11.

[16] Robert, L. (1993). Pathogénie de l'athérosclérose. In: Athérosclérose., p. 49. Sandoz (France).

[17] Marquié, G., Petkov, P., and Duhault, J. (1980) Diabetic syndrome in sand rat (*Psammomys obesus*) with special reference to the pancreas. *Atherosclerosis,* **47,** 7–17.

[18] Marquié, G., Duhault, J., Hadjiisky, P., Petkov, P., and Bouissou, H. (1991) Diabetes mellitus in sand rat (*Psammomys obsus*): Microangiopathy during development of the Diabetic Syndrome. *Cell. Mol. Biol.,* **37,** 651–667.

[19] Marquié, G., Duhault, J., and Jacotot, B. (1984) Diabetes mellitus in sand rats (*Psammomys obesus*). Metabolic pattern during development of the diabetic syndrome. *Diabetes,* **33,** 438–443.

[20] Aouichat Bouguerra, S., Bourdillon, M. C., Dahmani, Y., and Bekkhoucha, F. (2001) Non insulin dependent diabetes in sand rat (*Psammomys obesus*) and production of collagen in cultured aortic smooth muscle cells. Influence of insulin. *Int. J. Exp. Diab. Res.,* **2,** 37–46.

[21] Daly, M., and Daly, S. (1973) On the ecology of *Psammomys obesus* (*Rodentia gerbillidae*) in the wadi saoura Algeria. *Mammalia,* **37,** 546–561.

[22] Salé, F. O., Marshesini, S., Fishman, P. H., and Berra, B. (1984) A sensitive enzymatic essay for determination of cholesterol in lipids extracts. *Anal. Biochem.,* **147,** 347–350.

[23] Folch, J., Lees, M., and Stanly, G. H. S. (1957) A simple method for the isolation and purification of total lipids from animal tissues. *J. Biol. Chem.,* **226,** 497–509.

[24] Peterkofsky, B., Chojkier, M., and Batman, J. (1982) Determination of collagen synthesis in tissue and cell culture systems. In: *Immunochemistry of the Extracellular Matrix*, *2. Application*, edited by Furthmayer, M. D., pp. 19–48. Boca Raton, FL, CRC Press.

[25] Laemmli, U. K. (1970) Cleavage of structural proteins during the assemblay of the head of bacteriophage T4. *Nature,* **227,** 680–685.

[26] Campbell, J. H. Campbell, G. R., Kocher, O., and Gabbiani, G. (1987) Cell biology of smooth muscle in culture: Implications for atherogenesis. *Int. Angiol.,* **6,** 73–79.

[27] Bourdillon, M. C., Dussère, E., Covacho, C., and Berthezène, F. (1991) Modulation des cellules musculaires artérielles en culture et mouvements de cholestérol. *Ann. Endocrinol.,* **52,** 468–466.

[28] Dussère, E., Bourdillon, M. C., Ciavatti, M., Covacho, C., and Renaud, S. (1993) Lipids biosynthesis in cultured arteriel smooth muscle cells is related to their phenotype. *J. Lipids,* **28,** 589–592.

[29] Tabacick, C., Valentin, J. P., Alian, S., and Descomps, B. (1991). Active cholesterol biosynthesis in cultured aortic smooth muscle cells: Evolution during the life span of the culture. *Atherosclerosis,* **86,** 123–137.

[30] Oram, J. F., Chait, A., and Bierman, E. L. (1987) Lipoprotein and cholesterol metabolism in cultured arterial smooth muscle cells. In: *Vascular Smooth Muscle in Culture*, edited by Campbell, J. H., and Gordon, H., pp. 62–76. Boca Raton, FL, CRC Press.

[31] Minors, L. K., Rothblat, G. H., and Glick, J. M. (1989) Triglycerides and cholesteryl ester hydrolysis in a cell model of smooth muscle foam cells. *J. Lipids Res.,* **30,** 189–197.

[32] Dussère, E., Bourdillon, M. C., Pulcini, T., and Berthezène, F. (1994) Decrease in high density lipoprotein binding sites is associated with decrease in intracellular cholesterol efflux in dedifferentiated aortic smooth muscle cells. *Biochim. Biophys. Acta,* **1212,** 235–244.

[33] Sandrs, M. (1994) The molecular and cellular concepts in atherosclerosis. *Pharmacol. Ther.,* **61** 109–153.

[34] Ang, A. H., Tachas, G., Campbel, J. H., Batman, J. F., and Campbell, G. R. (1990) Collagen synthesis by cultured rabbit aortic smooth muscle cells. Alteration with phenotype. *Biochem. J.,* **265,** 461–469.

[35] Majors, A. K., and Ehrhart, L. A. (1992) Cell density and proliferation modulate collagen snythesis and procollagen mRNA levels in arterial smooth muscle cells. *Exp. Cell Res.,* **200,** 168–174.

[36] Sakata, N., Meng, J., Jimi, S., Segawa, M., and Takebayashi, S. (1995) Aging of aorta and atherosclerosis role of non enzymatic glycation of collagen. *Nippon Ronen. Igakkai. Zasshi.,* **32,** 336–343.

[37] Karim, M. A., Miller, D. D., Farrar, M. A., Eleftheriades, E., Reddy, B. H., Brelan. C. M., and Samarel, A. M. (1995) Histomorphometric and biochemical correlates procollagen gene expression during vascular repair after experimental angioplasty. *Circulation,* **91,** 2049-2057.

[38] Nahman, N. S., Leonhart, K. L., Cosio, G., and Hebert, C. L. (1992) Effects of high glucose on cellular proliferation and fibronectin production by cultured human mesangial cells. *Kidney Int.,* **41,** 396–402.

[39] Benazzoug, Y., Borchiellini, C., Labat-Robert, J., Robert, L., and Kern, P. (1998) Effect of high glucose concentrations on the expressions of collagens and fibronectin by fibroblasts in culture. *Exp. Gerontol.,* **33,** 445–455.

[40] Robert, L., Jacob, M. P., and Labat-Robert, J. (1992) Cell matrix interactions in the genesis of atherosclerosis and atheroma. Effect of aging. *Ann. N. Y. Acad. Sci.,* **673,** 333–341.

[41] Ahmed, N. (1991) Glycation and diabetic complications. *J. P. M. A.,* **7,** 171–174.

[42] Lyons, T. (1993) Glycation and oxidation. A role in the pathogenesis of atherosclerosis. *Am. J. Cardiol.,* **71,** B26–B31.

[43] Haraki, N., Higashi, T., Mori, T., Shibayama, R., Kawabe, Y., Kodama, T., Takahashi, K., Shichiri, M., and Horiuchi, S. (1995) Macrophage scavenger receptor mediates uptake and degradation of advanced glycation end products of the maillard reaction. *Eur. J. Biochem.,* **230,** 408–415.

